# Transcriptomic profiling analysis of human endometrial stromal cells treated with autologous platelet‐rich plasma

**DOI:** 10.1002/rmb2.12498

**Published:** 2023-01-22

**Authors:** Keiji Kuroda, Akemi Matsumoto, Takashi Horikawa, Satoru Takamizawa, Asako Ochiai, Kazuhiro Kawamura, Koji Nakagawa, Rikikazu Sugiyama

**Affiliations:** ^1^ Center for Reproductive Medicine and Endoscopy Sugiyama Clinic Marunouchi Tokyo Japan; ^2^ Centre for Reproductive Medicine and Implantation Research Sugiyama Clinic Shinjuku Tokyo Japan; ^3^ Department of Obstetrics and Gynaecology Juntendo University Faculty of Medicine Tokyo Japan

**Keywords:** cell growth, decidualization, endometrium, phosphoinositide 3‐kinase signaling pathway, platelet‐rich plasma

## Abstract

**Purpose:**

To clarify the mechanisms of intrauterine platelet‐rich plasma (PRP) infusion that support embryo implantation in in vitro fertilization treatment.

**Methods:**

Blood and endometrial samples were collected from four infertile women. Human endometrial stromal cells (HESCs) were cultured and passaged equally into four cell culture dishes in each patient. Two were treated with PRP twice, and the other two were treated with vehicle. Subsequently, two cultures with and without PRP were decidualized with 8‐bromoadenosine 3′,5′‐cyclic AMP and progesterone for 5 days.

**Results:**

The gene expression in undifferentiated or decidualized HESCs with and without PRP was compared. In the microarray analysis, 381 and 63 differentially expressed genes were detected in undifferentiated and decidualized HESCs, respectively. In the undifferentiated HESCs, PRP was found to promote the gene expression associated with cell growth, tissue regeneration, proinflammatory response, and antibiotic effects. In decidualized HESCs, PRP was found to attenuate the gene expression involved in cell proliferation and inflammation by inhibiting the expression of phosphoinositide 3‐kinase signaling.

**Conclusions:**

Platelet‐rich plasma regulates the reprogramming of cell proliferation and inflammation depending on menstrual cycle phases in an appropriate manner, suggesting that PRP has the potential to increase endometrial thickness in the proliferative phase and improve immune tolerance in the secretory phase.

## Graphical abstract

Intrauterine platelet‐rich plasma (PRP) infusion promotes tissue repair with transient inflammatory response, cell growth and migration, and antibiotic effect in undifferentiated human endometrial stromal cells, whereas cell proliferation and the inflammatory immune response are attenuated during decidualization of the endometrium. Depending on the menstrual cycle phases, PRP can regulate the reprogramming of inflammation and cell proliferation in an appropriate manner to support embryo implantation.

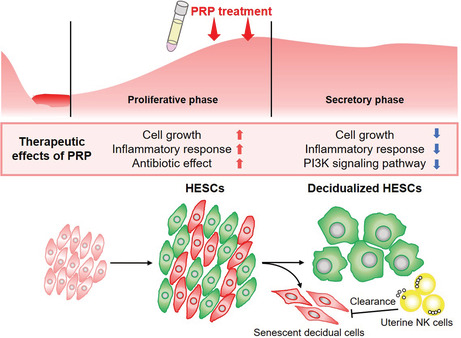

## 1 INTRODUCTION

Although there has been advancement in the field of assisted reproductive technology and chromosomal analysis of embryonic aneuploidy is being performed worldwide, the use of elective euploid embryos does not result in a successful pregnancy in all patients with a history of repeated implantation failure (RIF).^1^, ^2^ Embryo implantation requires not only embryo competence but also endometrial receptivity and maternal immune tolerance.^3^ Human endometrial cells proliferate with increasing estrogen secretion and are transformed into decidual cells via the stimulation of progesterone secreted from the corpus luteum. In this process, the decidualizing endometrium acquires receptivity to a semiallograft or a complete allograft embryo with optimal local angiogenesis and trophoblast invasion at implantation.^4^

Ultrasonography is used in clinical in vitro fertilization (IVF) treatment to evaluate whether the endometrium of the patient is stable for implantation. A thin endometrium is reportedly one of the prognostic factors of a low chance of conceiving after embryo transfer.^5^ A thin endometrium is primarily the result of intrauterine damage and adhesions after mechanical manipulations, including endometrial curettage and hysteroscopic surgery. The human endometrium is regenerated cyclically with the menstrual cycle because the endometrial stem cells contribute significantly to the proliferation and decidualization of the endometrium.^6^, ^7^ Therefore, the main target of treatment for a thin endometrium is the regeneration of mesenchymal stem cells, such as endometrial infusion of granulocyte colony‐stimulating factor.^8^, ^9^ However, the efficacy of this therapy remains controversial.^10^

Platelet‐rich plasma (PRP) has been utilized in various clinical areas, including orthopedics, ophthalmology, and dermatology.^11^, ^12^, ^13^ In the process of tissue repair, platelets are activated for the purpose of regeneration with inflammation, cellular migration, extracellular matrix remodeling, cell proliferation, differentiation, and angiogenesis.^14^, ^15^, ^16^ Platelets also have antimicrobial activity with the secretion of antimicrobial peptides.^17^ Thus, PRP is used at the site of tissue injury for repair and pain relief in various pathologies. In gynecology, administration of PRP has been recently introduced to treat impaired endometrial and ovarian functions in infertile women.^18^ Many studies have reported the therapeutic effects of intrauterine PRP infusion with increasing endometrial thickness and chance of embryo implantation.^19^, ^20^, ^21^, ^22^, ^23^, ^24^, ^25^ Thus, PRP is a widely used treatment for infertile women. However, the mechanisms underlying the improvement in endometrial receptivity remain poorly understood. In this study, the molecular influences of PRP treatment on human endometrial and decidualized cells were studied.

## 2 MATERIALS AND METHODS

### 2.1 Patient selection

This study was approved by the local ethics committee of Juntendo University (No. 20‐178) and Sugiyama Clinic (No. 19‐005). All experiments were performed in accordance with the relevant guidelines and regulations. With regard to the inclusion criteria, patients in the age range of 20–40 years with a history of four or more embryo transfer cycles were selected. Patients who used anticoagulant drugs and smoked were excluded. Of the five infertile women selected between August and September 2020, four were included after excluding one woman because of the insufficient amount of RNA collected. Table S1 presents the patients' characteristics. None of the patients had chronic endometritis (CE) resulting from CD138 immunostaining of the endometrium or abnormal complete blood count data. The diagnosis of CE was defined as five or more CD138‐positive cells in 10 random areas at ×10 magnification based on our previous studies.^26^, ^27^

### 2.2 Endometrial and blood sampling

All participating subjects provided written informed consent before sample collection. Endometrial sampling was performed using an endometrial suction curette (Pipet Curet; Fuji Medical Corporation) in 7–10 days following the luteinizing hormone surge. On the same day, 60 ml of blood sample was collected slowly using an 18‐gauge needle and transferred into a blood transfusion pack containing the anticoagulant citrate phosphate dextrose adenine (CPDA; Terumo Blood Bag CPDA, Terumo Corporation), which was mixed gently and kept in cold storage for 4–6 days until PRP treatment.

### 2.3 PRP preparation

On the day of cell treatment with PRP, approximately 30 ml of blood was drawn from the blood transfusion pack and processed using a PRP preparation system (SmartPrep® 3; Terumo Bct. Inc.) according to the manufacturer's instructions. The whole blood sample was added to the first chamber, and the centrifuge was activated. The red blood cells and the PRP were separated. The PRP was automatically decanted into the second chamber and concentrated by centrifugation. After platelet‐poor plasma was removed, 3 ml of the PRP was collected.

### 2.4 Primary culture of human endometrial stromal cells

Collected human endometrial stromal cells (HESCs) were isolated, cultured, and maintained as described previously.^28^, ^29^ The endometrial samples were obtained in Dulbecco's modified Eagle's medium (DMEM)–Ham's F‐12 (Nacalai Tesque) containing 1% (v/v) antibiotic solution, which was minced finely and digested enzymatically with total 5 mg collagenase (500 μg/ml; Sigma‐Aldrich) and 1 mg deoxyribonuclease (DNase) type I (100 μg/ml; Roche Applied Science) for 1 h at 37°C. After centrifugation, the cells were suspended in DMEM/F12 culture media containing 10% (v/v) dextran‐coated charcoal (DCC)‐treated fetal bovine serum, 1% antibiotic solution, 1% (v/v) l‐glutamine, 0.2% (v/v) insulin, and 1 nM estradiol (all from Sigma‐Aldrich).

We conducted the experiments to reproduce the clinical protocol for PRP treatment in vitro.^19^, ^20^, ^23^, ^24^, ^25^ Endometrial cells were cultured until they achieved confluency in 75‐cm^2^ culture flasks at 37°C in 5% carbon dioxide and then passaged equally into four 1‐well cell culture dishes (100 × 20 mm). After the HESCs were grown to approximately 80% confluence, a cell‐free strip as the cell proliferation zone was created by scratching using a 200‐ml pipette tip. Assuming clinical intrauterine PRP infusion, two cell culture dishes were treated and cultured with 1 ml of PRP each for 24 h (media containing 10% of PRP in total) on 1 and 3 days after endometrial scratching. The other two dishes were cultured with media without PRP. The 10% of PRP concentration in the culture media was determined based on previous clinical studies using 1 ml of PRP^19^, ^20^, ^22^, ^23^, ^25^ and approximately 10 cm^3^ of an average intrauterine volume in adult women with 57 mm of uterine length, 34 mm of width, and 8–10 mm of endometrial thickness.^30^ Four days after scratching, two culture dishes with and without PRP treatment were maintained in DMEM/F12 without phenol red (Thermo Fisher Scientific) containing 2% (v/v) DCC‐FBS and treated with 0.5 mM 8‐bromoadenosine 3′,5′‐cyclic adenosine monophosphate (8‐bromo‐cAMP) and 1 μM progesterone (P4) for decidualization. The other two culture dishes were cultured in media without 8‐bromo‐cAMP and P4. All culture samples were harvested 9 days after scratching (5 days of decidualization).

### 2.5 Cell proliferation assay

To confirm the effects of PRP on cell proliferation, the images of primary cultures of HESCs were acquired at 400‐fold magnification using an All‐in‐one Fluorescence Microscope (BZ‐X800; KEYENCE Corporation) on the day of scratching, after 1 day of scratching (the day of PRP treatment), and after 3 days of scratching (2 days after PRP treatment). The images were modified into phase contrast images of the cell proliferation zone using Image J 1.53k software (Wayne Rasband and contributors, National Institutes of Health). The number of endometrial cells in the strip was then counted.

### 2.6 RNA extraction and real‐time quantitative polymerase chain reaction (RT‐qPCR)

Total RNA was extracted from primary HESC cultures using the RNeasy plus mini kit (QIAGEN). cDNA was generated using the SuperScript II Reverse Transcriptase for RT‐qPCR kit (Thermo Fisher Scientific) and performed template quantification using the 7500 fast RT‐qPCR system (Thermo Fisher Scientific) with a dye layer, power SYBER Green PCR Master Mix (Thermo Fisher Scientific). RNA input variances were normalized against the levels of the housekeeping gene, *L19*, which encodes a ribosomal protein. All measurements were performed in duplicate or triplicate. Specific primer pairs were used as follows: L19 sense, 5′‐GCG GAA GGG TAC AGC CAA T‐3′, L19‐R antisense, 5′‐GCA GCC GGC GCA AA‐3′; decidual prolactin (PRL) sense, 5′‐AAG CTG TAG AGA TTG AGG AGC AAA C‐3′, decidual PRL antisense, 5′‐TCA GGA TGA ACC TGG CTG ACT A‐3′; insulin‐like growth factor binding protein 1 (IGFBP1) sense, 5′‐CGA AGG CTC TCC ATG TCA CCA‐3′, IGFBP1 antisense, 5′‐TGT CTC CTG TGC CTT GGC TAA AC‐3′. The RT^2^ Profiler TaqMan® PCR Array, Human PI3K‐AKT Signaling Pathway (330 231 PAHS‐058ZA, QIAGEN) was used to identify detailed PRP‐affected genes in decidualized HESCs. SYBR‐Green RT‐qPCR was performed using synthesized cDNA from the pooled RNA. This array includes 84 genes in members of the PI3K/AKT families and their regulators. Each measurement was performed from primary cultures with and without PRP in the decidualized cells of four infertile patients in duplicate. RNA input variances were normalized against the levels of the *GAPDH* housekeeping gene.

### 2.7 Microarray and gene ontology analyses

Isolated total RNA was amplified and labeled using the GeneChip WT PLUS Reagent Kit (Thermo Fisher Scientific) according to the manufacturer's manual. First, total RNA (100 ng) was primed with primers containing a T7 promoter sequence and converted into single‐stranded cDNA with T7 promoter sequence at the 5′ end. Using DNA polymerase and RNase H, single‐stranded cDNA was converted to double‐stranded cDNA. Antisense RNA (complementary RNA: cRNA) was synthesized and amplified by in vitro transcription of the second‐stranded cDNA template using T7 RNA polymerase. Enzymes, salts, inorganic phosphates, and unincorporated nucleotides were removed to purify the cRNA, and the concentration of cRNA was calculated using NanoDrop ND‐2000 (Thermo Fisher Scientific). Sense‐strand cDNA containing dUTP at a fixed ratio relative to dTTP was synthesized by the reverse transcription of cRNA using second‐cycle primers. After hydrolysis of the cRNA template using RNase H, the second‐cycle single‐stranded cDNA was purified, and the concentration was calculated. The purified, sense‐strand cDNA (5.5 μg) was fragmented by uracil‐DNA glycosylase and apurinic/apyrimidinic endonuclease1 at the unnatural dUTP residues and broke the DNA strand. Using the proprietary DNA‐labeling reagent, the fragmented cDNA was labeled by terminal deoxynucleotidyl transferase and hybridized to GeneChip Human Gene 2.0 ST Array (Thermo Fisher Scientific). The array was incubated for 16 h at 45°C and then automatically washed and stained with GeneChip Hybridization, Wash and Stain Kit (Thermo Fisher Scientific). After the array was scanned using a GeneChip Scanner 3000 7G, the data were analyzed using GeneChip Command Console Software and Expression Console. Data were summarized according to the robust multichip analysis (RMA) algorithm. The microarray data sets used in the present study were deposited in the Gene Expression Omnibus under the accession number of GSE213873 (https://www.ncbi.nlm.nih.gov/geo/query/acc.cgi?acc=GSE213873).

Further analyses including principal component analysis were conducted on the 48 144 probes available for the technology (Affymetrix, HuGene‐2_0‐st_na36_hg19, 2016.7.6) using GeneSpring GX software version 14.9.1 (Agilent Technologies). Using the RMA algorithm, summarized intensity values were submitted to the baseline transformation algorithm in which the median of the log summarized values from the control samples was first computed and then subtracted from the sample. Altered transcripts were identified using a comparative method on the baseline transformed intensity values between the “control” and “sample” probes. The genes corresponding to the probes that had a change in intensity exceeding a ratio of 2.0 were considered as genes with a significant differential expression pattern. In parallel, an unpaired *t*‐test with unequal variance (Welch's *t*‐test) including false discovery rate filter was performed on the totality of the probes to compare the means of the two groups of replicates. Probes with *p* < 0.05 were considered to have significantly different signal value means. Gene ontology (GO) analysis was performed on the sets of probes that were common to both groups. The ontologies with a corrected *p* < 0.1 were considered significant. *Z*‐score hierarchical clustering heatmaps were created using heatmap analysis software, *Heatplus (annHeatmap2) of* R package version 3.4.0.

### 2.8 Statistical analysis

Results are reported as mean ± standard deviation or standard error of the mean. Statistical analysis was performed using one‐way analysis of variance followed by the Wilcoxon signed‐rank test in the cell proliferation array and the Mann–Whitney *U*‐test in the PCR array of human PI3K‐AKT signaling pathway, following normalization of the data with GraphPad Prism 5 (GraphPad Software Inc.). The level of significance was defined as *p* < 0.05.

## 3 RESULTS

### 3.1 Proliferation of HESCs with or without PRP treatment

**FIGURE 1:**
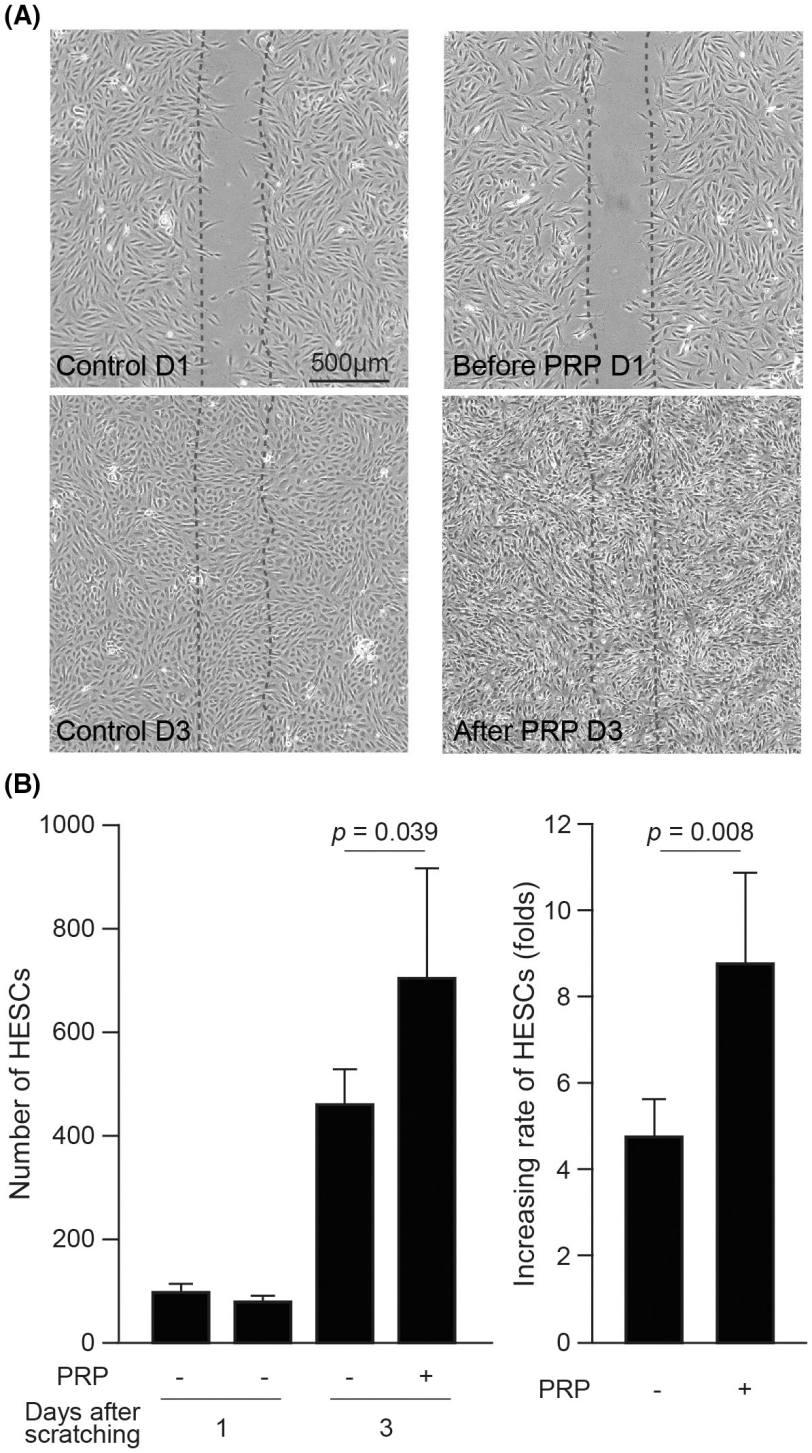
Cell proliferation in undifferentiated human endometrial stromal cells (HESCs) with and without platelet‐rich plasma (PRP) treatment. (A) Cell proliferation in the strips after endometrial scratching in undifferentiated HESCs treated with and without PRP. (B) Number and increasing rate in the strips in eight independent primary cultures from four patients at 1 and 3 days after endometrial scratching. The data are expressed as mean fold change ± SEM. The dates are counted from the day of endometrial scratching.

To examine cell proliferation of HESCs treated with or without PRP, a cell‐free strip as the cell proliferation zone was created by endometrial scratching, and the migrated cells in the strip on 1 and 3 days after endometrial scratching (before and 2 days after PRP treatment, respectively) were counted (Figure 1). The migrated cells had reached confluency in all strips on the third day after endometrial scratching. In HESCs without PRP treatment, the numbers of cells in the strips on the first and third days after scratching were 97.9 ± 16.3 and 460.0 ± 60.7 cells, respectively. In the HESCs treated with PRP, the cell counts before and after PRP treatment were 79.4 ± 11.9 and 704.4 ± 211.9 cells, respectively. The number of cells treated with PRP after 3 days was significantly larger than that treated without PRP (*p* = 0.039). The periphery of the cells that underwent PRP treatment appeared whitish with high cell confluency (Figure 1A, lower right panel). The growth rate of HESCs treated with PRP was higher than that of HESCs without PRP treatment (8.8 ± 2.1‐fold and 4.8 ± 0.9‐fold, respectively, *p* = 0.008; Figure 1B).

### 3.2 Identification of genes affected by PRP in undifferentiated and decidualized HESCs

**FIGURE 2:**
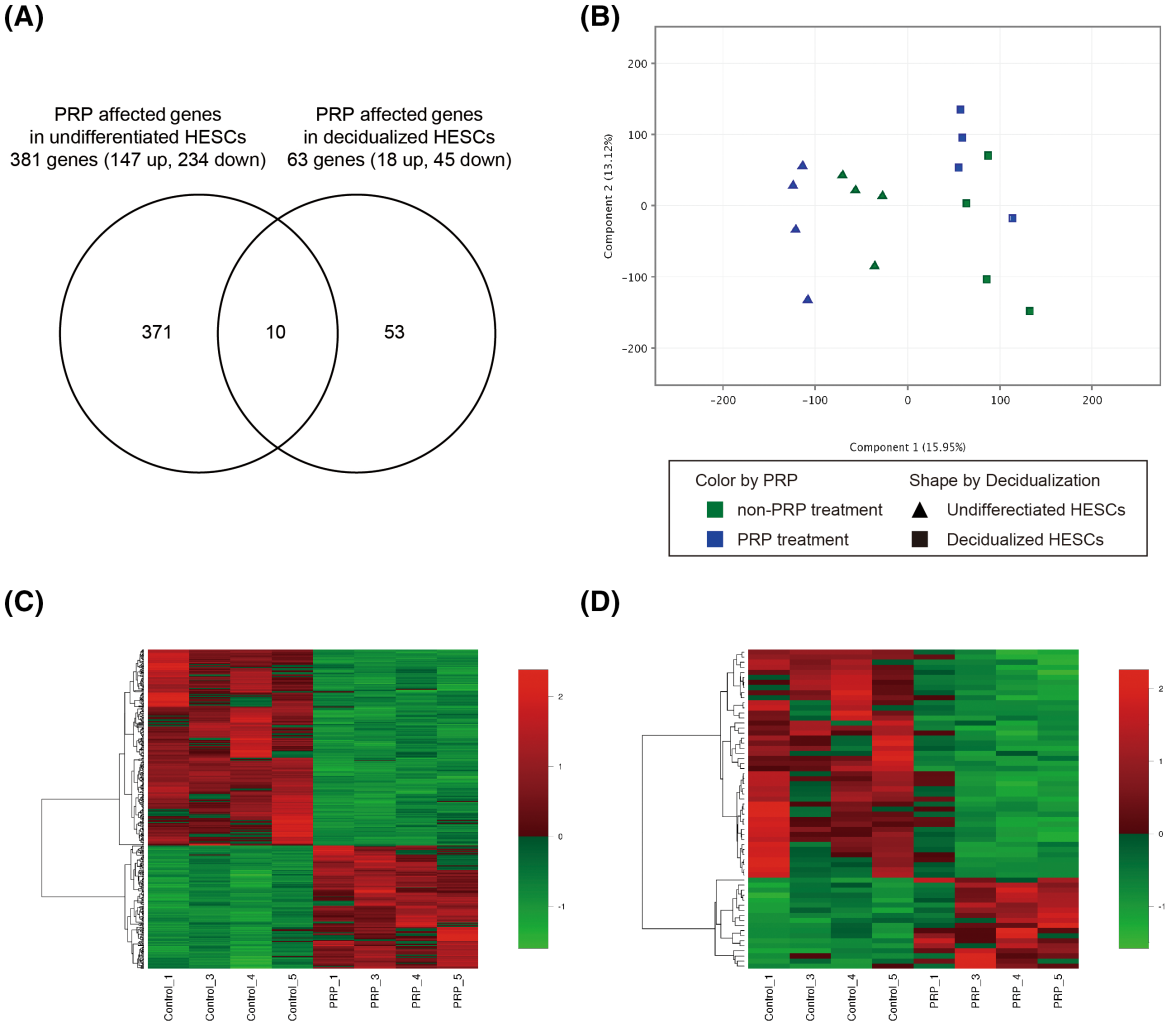
Platelet‐rich plasma (PRP)‐affected genes in undifferentiated and decidualized human endometrial stromal cells (HESCs). (A) Venn diagram showing 381 and 63 PRP‐affected genes were extracted in undifferentiated and decidualized HESCs, respectively. Ten genes were identified in both undifferentiated and decidualized HESCs. (B) Principal component analysis in undifferentiated and decidualized HESCs with and without PRP. Blue and green plots show the samples with and without PRP treatment, respectively. Triangle and square plots show the samples of undifferentiated and decidualized HESCs, respectively. (C) Heatmap analysis in undifferentiated HESCs with and without PRP (D) Heatmap analysis in decidualized HESCs with and without PRP.

In order to identify the genes affected by PRP treatment in undifferentiated and decidualized HESCs, four individual primary cultures were prepared in each patient. Two cultures were treated with PRP 1 and 3 days after endometrial scratching, and the other two cultures were not treated with PRP. Subsequently, two cultures with and without PRP were decidualized with 8‐bromo‐cAMP and P4 for 5 days (decidualized HESCs with and without PRP). The other two cultures with and without PRP were not decidualized (undifferentiated HESCs with and without PRP). After morphologically confirming the presence/absence of decidual change in the HESCs treated with 8‐bromo‐cAMP and P4, the total mRNA from four individual primary cultures with or without PRP treatment in undifferentiated or decidualized HESCs were extracted for RT‐qPCR and whole‐genome microarray analysis. To confirm the impact of PRP on the decidualization of HESCs, the mRNA expression levels of decidual markers, *PRL* and *IGFBP1*, were compared in decidualized HESCs treated with and without PRP treatment. In comparison with the decidualized cells without PRP, the addition of PRP downregulated the expression levels of *PRL* and *IGFBP1* (*p* = 0.029 and 0.125, respectively; Figure S1). Using a cutoff value of ≥2.0‐fold change in the microarray analysis, 381 and 63 PRP‐affected genes were extracted in undifferentiated and decidualized HESCs, respectively (Figure 2A, Table S2). Ten genes were identified in both undifferentiated and decidualized HESCs (Table S3). Abundant genes in the undifferentiated HESCs were found to be affected by PRP; however, the effect of PRP may have been attenuated in decidualized cells. PRP treatment resulted in more downregulated than upregulated genes in both undifferentiated and decidualized HESCs (234 vs. 147 in undifferentiated HESCs and 45 vs. 18 in decidualized HESCs, respectively). Figure 2B–D shows a principal component analysis and *z*‐score hierarchical clustering heatmaps in undifferentiated or decidualized HESCs with and without PRP.

### 3.3 PRP enhances gene expressions associated with tissue repair, proinflammatory immune response, and antimicrobial effect in undifferentiated HESCs

Gene ontology analysis detected various enriched GO terms of statistical significance in 381 genes affected by PRP in undifferentiated HESCs (Table 1). In the upregulated genes, the top 10 GO terms included regulation of immune effector process, nucleosome, protein‐DNA complex, negative regulation of biological process, chromosome, regulation of leukocyte‐mediated immunity, positive regulation of cell migration, positive regulation of defense response to virus by host, regulation of T‐helper 1 (Th1) type immune response, and positive regulation of cellular component movement. In addition, other GO terms indicated that PRP treatment was associated with tissue remodeling (including the regulation of angiogenesis, cell proliferation, differentiation, cell cycle, and cell adhesion), proinflammatory immune (Th1 and T‐helper 17 cells [Th17]) response (including the regulation of tumor necrosis factor [TNF] superfamily cytokine, interleukin [IL]‐1 receptor, IL‐10, IL‐17, and interferon‐gamma [INF‐γ]), and defense response to infection (including the regulation of neutrophil chemotaxis and defense response to gram‐negative bacterium and granulocyte‐macrophage colony‐stimulating factor production). Table 2 shows the genes upregulated in undifferentiated HESCs upon treatment with PRP. PRP showed a remarkable potential for wound healing and protection against infection in undifferentiated HESCs.

**TABLE 1:** Gene ontology.

GO term	Count	*p*‐Value
GO terms enriched among 147 upregulated genes in undifferentiated HESCs treated with PRP (undifferentiated HESCs without PRP vs. undifferentiated HESCs with PRP)		
Regulation of immune effector process	19	2.32E‐10
Nucleosome	11	2.59E‐10
Protein‐DNA complex	13	3.10E‐10
Negative regulation of biological process	69	9.45E‐10
Chromosome	26	1.71E‐09
Regulation of leukocyte‐mediated immunity	12	4.09E‐09
Positive regulation of cell migration	17	5.80E‐09
Positive regulation of defense response to virus by host	11	9.34E‐09
Regulation of T‐helper 1 type immune response	7	1.31E‐08
Positive regulation of cellular component movement	17	1.20E‐08
GO terms enriched among 234 downregulated genes in undifferentiated HESCs treated with PRP (undifferentiated HESCs without PRP vs. undifferentiated HESCs with PRP)		
System development	100	1.29E‐13
Nervous system development	69	1.32E‐12
Multicellular organism development	106	1.89E‐12
Anatomical structure development	109	9.85E‐12
Homophilic cell adhesion via plasma membrane adhesion molecules	16	4.25E‐11
Cell–cell adhesion via plasma membrane adhesion molecules	18	7.14E‐11
Biological adhesion	36	7.17E‐11
Synapse assembly	11	7.59E‐11
Developmental process	111	1.09E‐10
Calcium‐dependent cell–cell adhesion via plasma membrane cell adhesion molecules	10	1.83E‐10
GO terms enriched among 15 upregulated genes in decidualized HESCs treated with PRP (decidualized HESCs without PRP vs. decidualized HESCs with PRP)		
Negative regulation of phosphatidylinositol 3‐kinase (PI3K) signaling	2	3.59E‐05
Regulation of response to wounding	3	1.37E‐04
Mating behavior	2	1.49E‐04
Reproductive behavior	2	2.72E‐04
Long‐term synaptic potentiation	2	4.91E‐04
GO terms enriched among 38 downregulated genes in decidualized HESCs treated with PRP (decidualized HESCs without PRP vs. decidualized HESCs with PRP)		
Proteinaceous extracellular matrix	8	9.40E‐07
Nitric oxide production involved in inflammatory response	2	4.38E‐06
Regulation of multicellular organismal development	13	3.45E‐05
Regulation of cell growth	6	2.89E‐04
Collagen binding	3	4.02E‐04

**TABLE 2:** Top 50 genes upregulated in undifferentiated human endometrial stromal cells upon treatment with platelet‐rich plasma.

Gene symbol	Gene name	Fold change
CGA	Glycoprotein hormones, alpha polypeptide	8.77
ATP6V0D2	ATPase H+ transporting V0 subunit d2	8.77
IL1B	Interleukin 1 beta	7.05
MT1M	Metallothionein 1M	6.77
MT1L	Metallothionein 1L, pseudogene	6.77
HAS2	Hyaluronan synthase 2	6.06
ACPP	Acid phosphatase, prostate	5.36
PLEK2	Pleckstrin 2	5.23
SERPINB2	Serpin family B member 2	5.00
SERPINB10	Serpin family B member 10	5.00
CXCL8	C‐X‐C motif chemokine ligand 8	4.79
TRBV27	T cell receptor beta variable 27	4.66
VEPH1	Ventricular zone expressed PH domain containing 1	4.46
TRHDE	Thyrotropin‐releasing hormone‐degrading enzyme	4.31
IL1A	Interleukin 1 alpha	4.13
FOXF1	Forkhead box F 1	4.11
TMEM158	Transmembrane protein 158	4.11
TMEM156	Transmembrane protein 156	3.99
PTGS1	Prostaglandin‐endoperoxide synthase 1	3.84
FAM180A	Family with sequence similarity 180 member A	3.80
TMEM244	Transmembrane protein 244	3.79
ARHGAP18	Rho GTPase activating protein 18	3.79
GPR37	G protein‐coupled receptor 37	3.78
MMP12	Matrix metallopeptidase 12	3.74
NPR3	Natriuretic peptide receptor 3	3.61
TRBV3‐1/TRBV7‐2/TRBV6‐5/TRBC2	T cell receptor beta variable 3‐1/T cell receptor beta variable 7‐2/T cell receptor beta variable 6‐5/T cell receptor beta constant 2	3.57
IL13RA2	Interleukin 13 receptor alpha 2	3.53
LNCOG	lncRNA osteogenesis associated	3.49
C10orf55	Chromosome 10 putative open reading frame 55	3.45
CYP2S1	Cytochrome P450 family 2 subfamily S member 1	3.41
LOC105376374	Uncharacterized LOC105376374	3.39
TM4SF1	Transmembrane 4 L six family member 1	3.36
DCSTAMP	Dendrocyte expressed seven transmembrane protein	3.33
F2RL1	F2R‐like trypsin receptor 1	3.32
CAMK1G	Calcium/calmodulin‐dependent protein kinase IG	3.31
KDR	Kinase insert domain receptor	3.31
PLAU	Plasminogen activator, urokinase	3.20
MMP1	Matrix metallopeptidase 1	3.19
IL7R	Interleukin 7 receptor	3.17
ENPP1	Ectonucleotide pyrophosphatase/phosphodiesterase 1	3.16
MYPN	Myopalladin	3.12
RPL23AP82	Ribosomal protein L23a pseudogene 82	3.09
STC1	Stanniocalcin 1	3.09
CST4	Cystatin S	3.04
STEAP1	STEAP family member 1	3.01
TNFSF15	TNF superfamily member 15	2.98
FAM20C	FAM20C golgi associated secretory pathway kinase	2.96
LPXN	Leupaxin	2.93
IL1RL1	Interleukin 1 receptor‐like 1	2.82

However, based on the GO terms among 234 downregulated genes, PRP attenuated the expression of genes associated with system development and cell adhesion (Table 1). Table 3 shows the top 50 genes downregulated in undifferentiated HESCs upon treatment with PRP.

**TABLE 3:** Top 50 genes downregulated in undifferentiated human endometrial stromal cells upon treatment with platelet‐rich plasma.

Gene symbol	Gene name	Fold change
ALDH1A1	Aldehyde dehydrogenase 1 family member A1	−12.38
ADRA2A	Adrenoceptor alpha 2A	−10.38
OLFML1	Olfactomedin‐like 1	−8.54
PLXDC2	Plexin domain containing 2	−8.09
SLC40A1	Solute carrier family 40 member 1	−6.68
CNTN1	Contactin 1	−6.62
TNFRSF19	TNF receptor superfamily member 19	−6.34
ABCA9	ATP‐binding cassette subfamily A member 9	−6.33
SESN3	Sestrin 3	−5.83
PCDH10	Protocadherin 10	−5.60
SCD	Stearoyl‐CoA desaturase	−5.54
ABCA6	ATP‐binding cassette subfamily A member 6	−5.52
ISM1	Isthmin 1	−5.16
PDGFD	Platelet‐derived growth factor D	−5.14
ABCA8	ATP‐binding cassette subfamily A member 8	−5.13
CFH	Complement factor H	−5.01
SEMA3D	Semaphorin 3D	−4.96
MFAP4	Microfibril associated protein 4	−4.93
PLXDC2	Plexin domain containing 2	−4.75
MYLIP	Myosin regulatory light chain interacting protein	−4.65
RASSF2	Ras association domain family member 2	−4.53
CXCL12	C‐X‐C motif chemokine ligand 12	−4.49
GXYLT2	Glucoside xylosyltransferase 2	−4.11
GBP2	Guanylate‐binding protein 2	−4.09
BRINP1	BMP/retinoic acid inducible neural specific 1	−4.01
TMTC1	Transmembrane O‐mannosyltransferase targeting cadherins 1	−3.92
GBP4	Guanylate‐binding protein 4	−3.90
FMOD	Fibromodulin	−3.78
PCYT1B	Phosphate cytidylyltransferase 1B, choline	−3.78
GBP2	Guanylate‐binding protein 2	−3.63
DLX5	Distal‐less homeobox 5	−3.60
ADGRL3	Adhesion G protein‐coupled receptor L3	−3.52
HSPB6	Heat shock protein family B (small) member 6	−3.49
LOC105377979	Uncharacterized LOC105377979	−3.44
PCDHB14	Protocadherin beta 14	−3.42
CEMIP	Cell migration inducing hyaluronidase 1	−3.38
WNT2	Wnt family member 2	−3.34
CNTN5	Contactin 5	−3.33
ADGRD1	Adhesion G protein‐coupled receptor D1	−3.26
RORB	RAR‐related orphan receptor B	−3.19
ECM2	Extracellular matrix protein 2	−3.14
DHRS3	Dehydrogenase/reductase 3	−3.14
ABLIM1	Actin‐binding LIM protein 1	−3.13
ANGPT1	Angiopoietin 1	−3.12
TMEM35	Transmembrane protein 35	−3.10
PRKAR2B	Protein kinase cAMP‐dependent type II regulatory subunit beta	−3.10
PTHLH	Parathyroid hormone‐like hormone	−3.08
JAKMIP2	Janus kinase and microtubule interacting protein 2	−3.08
PIK3R1	Phosphoinositide‐3‐kinase, regulatory subunit 1	−3.05
IGFBP3	Insulin‐like growth factor binding protein 3	−3.04

### 3.4 PRP regulates the phosphoinositide 3‐kinase/AKT signaling pathway in decidualized HESCs

**FIGURE 3:**
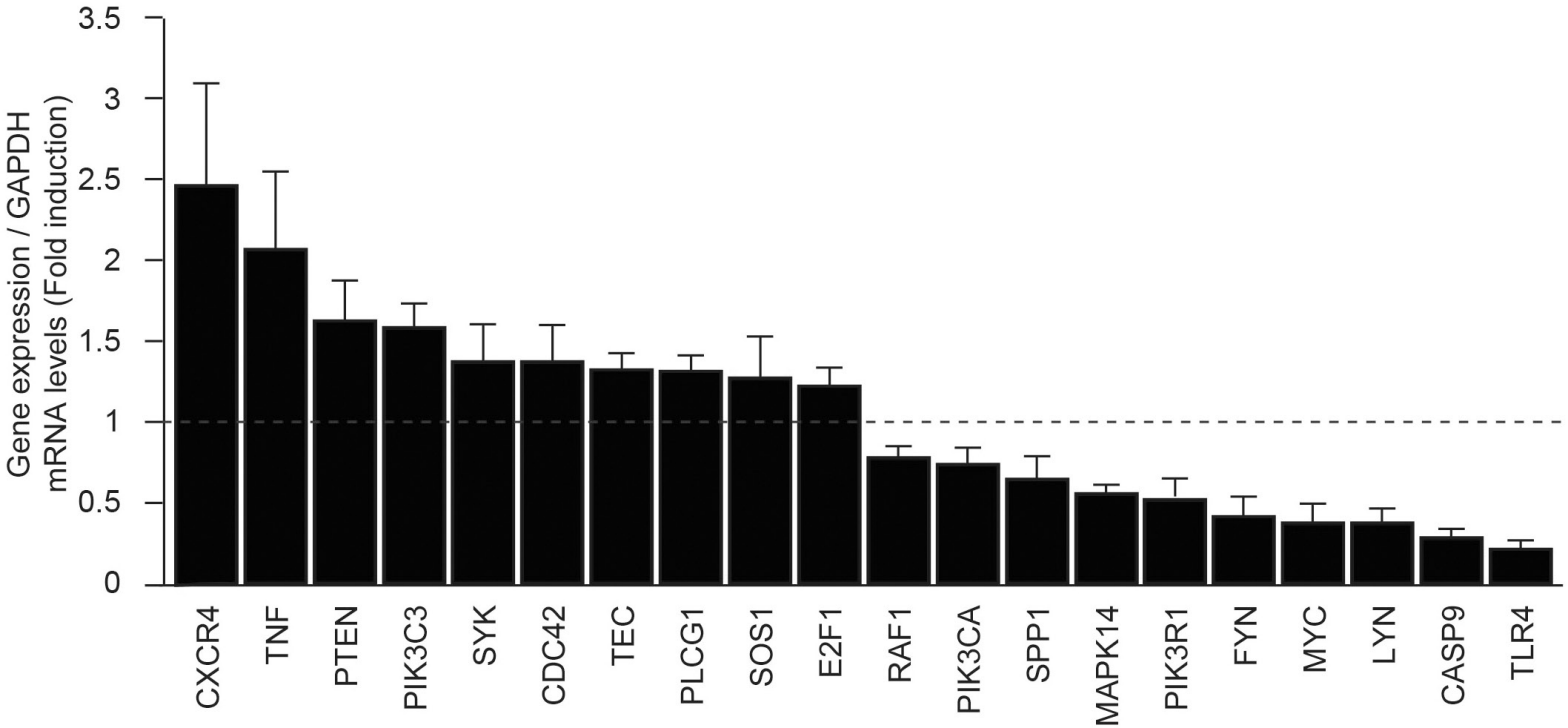
Platelet‐rich plasma (PRP)‐affected genes in the PI3K/AKT signaling pathway in decidualized human endometrial stromal cells (HESCs). In decidualized HESCs treated with and without platelet‐rich plasma, inclusive real‐time quantitative polymerase chain reaction (RTQ‐PCR) array analysis of the PI3K/AKT signaling pathway demonstrated 10 significantly upregulated genes and 10 significantly downregulated genes, respectively, using a cutoff value of *p* < 0.05.

In contrast to undifferentiated HESCs, the impact of PRP was limited with only 63 genes in the decidualized cells (Figure 2A). PRP treatment identified key genes involved in the PI3K signaling pathway, including *SERPINE2* (also referred to as protease nexin‐1: *PN1*) and *PTEN* as upregulated genes and *TLR4*, *DEPTOR*, and *RASGRP2* as downregulated genes (Tables 4 and 5). PI3K mediates AKT downstream signaling pathways of cellular proliferation, survival, and motility. Furthermore, the regulation of the PI3K/AKT signaling pathway is required for the process of decidual change in HESCs.^31^ Therefore, an inclusive RT‐qPCR array analysis was conducted in decidualized HESCs treated with and without PRP to identify detailed PRP‐dependent genes in the PI3K/AKT signaling pathway. Using a cutoff value of *p* < 0.05, the PCR array analysis showed 10 considerably upregulated genes, including *CXCR4*, *TNF*, *PTEN*, *PIK3C3*, *SYK*, *CDC42*, *TEC*, *PLCG1*, *SOS1*, and *E2F1*, and 10 significantly downregulated genes, including *RAF1*, *PIK3CA*, *SPP1*, *MAPK14*, *PIK3R1*, *FYN*, *MYC*, *LYN*, *CASP9*, and *TLR4* (Figure 3, Table S4).

**TABLE 4:** Genes upregulated genes in decidual human endometrial stromal cells upon treatment with platelet‐rich plasma.

Gene symbol	Gene name	Fold change
CDH12	Cadherin 12, type 2 (N‐cadherin2)	4.76
SULT1C4	Sulfotransferase family 1C member 4	2.73
PTCHD1	Patched domain containing 1	2.59
MIR1302‐5	microRNA 1302‐5	2.49
FBXL7	F‐box and leucine‐rich repeat protein 7	2.49
SERPINE2	Serpin family E member 2	2.44
TC2N	Tandem C2 domains, nuclear	2.38
CST4	Cystatin S	2.31
DPT	Dermatopontin	2.26
PLA2G4A	Phospholipase A2, group IVA	2.25
SEPTIN7P13	Septin 7 pseudogene 13	2.22
SNCAIP	Synuclein alpha interacting protein	2.18
ZNF521	Zinc finger protein 521	2.14
TRHDE	Thyrotropin‐releasing hormone‐degrading enzyme	2.10
LOC100131541	Uncharacterized LOC100131541	2.05
GUSBP3	Glucuronidase beta (GUSB) pseudo gene 3	2.05
LOC105379298	Uncharacterized LOC105379298	2.02
PTEN	Phosphatase and tensin homolog	2.00

**TABLE 5:** Genes downregulated genes in decidual human endometrial stromal cells upon treatment with platelet‐rich plasma.

Gene symbol	Gene name	Fold change
OMD	Osteomodulin	−8.60
SPARCL1	SPARC‐like 1	−4.74
IGFBP1	Insulin‐like growth factor binding protein 1	−4.46
IGFBPL1	Insulin‐like growth factor binding protein‐like 1	−4.24
CFH	Complement factor H	−3.89
SST	Somatostatin	−3.86
TLR4	Toll‐like receptor 4	−3.46
SIPA1L2	Signal‐induced proliferation‐associated 1‐like 2	−3.43
DEPTOR	DEP domain‐containing MTOR‐interacting protein	−3.34
TMEM132C	Transmembrane protein 132C	3.19
APOO	Apolipoprotein O	−3.07
CLEC2D	C‐type lectin domain family 2, member D	−3.05
ASPN	Asporin	−2.96
MGP	Matrix Gla protein	−2.91
SLC7A2	Solute carrier family 7, member 2	−2.77
SLC2A1	Solute carrier family 2, member 1	−2.74
ECM2	Extracellular matrix protein 2	−2.55
LINC00707	Long intergenic nonprotein coding RNA 707	−2.51
CDKN1C	Cyclin‐dependent kinase inhibitor 1C	−2.48
PYGM	Glycogen phosphorylase, muscle associated	−2.44
SORCS1	Sortilin‐related VPS10 domain‐containing receptor 1	−2.41
SH3GL2	SH3‐domain‐containing GRB2‐like 2, endophilin A1	−2.40
FAM89A	Family with sequence similarity 89, member A	−2.40
MIR1182	microRNA1182	−2.40
LSAMP	Limbic system‐associated membrane protein	−2.40
PODNL1	Podocan‐like 1	−2.37
RASGRP2	RAS guanyl‐releasing protein 2	−2.35
SLC10A6	Solute carrier family 10, member 6	−2.34
CMIP	c‐Maf inducing protein	−2.33
VSIG1	V‐set and immunoglobulin domain containing 1	−2.32
PLXNA4	Plexin A4	−2.31
EFEMP1	EGF containing fibulin extracellular matrix protein 1	−2.25
SRGN	Serglycin	−2.24
TMEM132B	Transmembrane protein 132B	−2.23
ACKR3	Atypical chemokine receptor 3	−2.19
ESYT3	Extended synaptotagmin protein3	−2.17
ATP8A2	ATPase aminophospholipid transporting 8A2	−2.16
RGCC	Regulator of cell cycle	−2.16
OXTR	Oxytocin receptor	−2.14
MCAM	Melanoma cell adhesion molecule	−2.13
LOC374443	C‐type lectin domain family 2 member D pseudogene	−2.12
OLFML2B	Olfactomedin‐like 2B	−2.10
SEMA3B	Semaphoring 3B	−2.06
SPHK1	Sphingosine kinase 1	−2.04
ODC1	Ornithine decarboxylase 1	−2.03
PXYLP1	2‐phosphoxylose phosphatase 1	−2.01

Contrary to the undifferentiated HESCs, PRP treatment downregulated the genes associated with cell growth regulation (*RASGRP2*, *SPHK1*, *SEMA3B*, *PLXNA4*, *IGFBP1*, and *IGFBPL1*) and inflammatory response (*SLC7A2* and *TLR4*) in decidualized HESCs.

## 4 DISCUSSION

In this study, the molecular impacts of PRP treatment on the human endometrium in a primary culture were identified first. In the proliferative phase, the exposure of estrogen in the endometrium increases endometrial thickness and the linear growth of endometrial glands and angiogenesis, which are processes that are indispensable for pregnancy. PRP was found to strongly promote cell proliferation and induce gene expressions associated with cell growth, including the proinflammatory response in undifferentiated HESCs. In another study, PRP was found to induce endometrial regeneration with cell proliferation in human endometrial cell lines,^32^ which was in agreement with our results. PRP containing abundant growth factors, such as transforming growth factor‐β, platelet‐derived growth factor, insulin‐like growth factor, vascular endothelial growth factor, and epidermal growth factor,^33^ is expected to accelerate cell growth. In addition, PRP treatment led to a considerably high expression of many histone genes (*HIST1H1A*, *HIST1H1B*, *HIST1H2A*, *HIST1H2A1*, *HIST1H2AB*, *HIST1H2AJ*, *HIST1H2BM*, *HIST1H3B*, *HIST1H3F*, *HIST1H3G*, *HIST1H3J*, and *HIST1H4D*) in undifferentiated HESCs (data not shown). An increase in acetylation levels of H2AK5, H3K9, and H4K8 is required to enable endometrial regeneration and proliferation in the early proliferative phase.^34^, ^35^ PRP may also induce histone acetylation for cell proliferation. In fact, all previous clinical studies have shown that intrauterine PRP infusion increases endometrial thickness in infertile women with thin endometria.^19^, ^20^, ^21^, ^22^, ^23^, ^24^, ^25^ PRP treatment might also affect cell migration, which should be investigated in future studies.

According to our results, PRP has a high potential to repair impaired endometrium such as in Asherman syndrome. In a murine model of Asherman syndrome, human PRP improved endometrial morphology and reduced fibrosis, resulting in an increased number of live births.^36^ Although PRP did not considerably improve postsurgical adhesion, endometrial thickness, and the pregnancy rate after hysteroscopic adhesiolysis in some randomized clinical trials with a small sample size,^37^, ^38^ some successful pregnancy cases have been reported.^39^ In our study, PRP demonstrated great potential for repairing a damaged intrauterine environment; thus, there is a need to reexamine the treatment protocol and conduct further clinical trials.

PRP also upregulated the genes involved in the beta chain of the T cell receptor (*TRBV3‐1*, *TRBV6‐5*, *TRBV7‐2*, *TRBV27*, *TRBC2*, and *TRBJ2‐3*), which is responsible for antigen recognition and activation of cellular immunity during bacterial infection.^40^ CE is a representative intrauterine disorder characterized by the continuous inflammatory status of the local endometrium with plasma cell marker CD138‐positive cells across different menstrual cycles. CE can cause implantation failure and pregnancy loss.^41^ Most cases of CE are caused by intrauterine infection with a wide variety of micro‐organisms; thus, the current treatment protocol for CE is broad‐spectrum antibiotic therapy.^42^ However, inappropriate systemic antibiotic therapy increases the risk of the emergence of antibiotic‐resistant bacteria and should be avoided.^43^ Intrauterine PRP infusion with antibiotic effects is expected to be used for the treatment of CE, instead of antibiotic therapy. Recently, some studies have already reported successful pregnancy outcomes after PRP infusion as a treatment for CE.^44^, ^45^ Further clinical trials on PRP treatment for CE are warranted in the future.

Platelet‐rich plasma influenced a large number of genes in undifferentiated HESCs, whereas in decidualized HESCs, the number of the genes affected by PRP was extremely as low as one‐sixth of the undifferentiated HESCs. It is expected that the effect of PRP on the endometrium reduces with culture time. In clinical practice, intrauterine PRP infusion affects the endometrium in the proliferative phase, yet it may have a weaker effect in the secretory phase. Intriguingly, PRP treatment both positively and negatively affected inflammation and cell proliferation in undifferentiated and decidualized HESCs, respectively. The function and physiology of endometrial development are completely different in the secretory phase (decidual phase) than that in the proliferative phase. Decidualization starts with an acute stress response with the secretion of proinflammatory mediators.^46^, ^47^ In the initial decidual phase, some stromal cells burdened by replication stress fail to differentiate as senescent decidual cells.^46^, ^47^ Senescent decidual cells secrete the senescence‐associated secretory phenotype, including proinflammatory cytokines and chemokines, thus inducing secondary senescence in adjacent decidual cells.^47^ Uterine natural killer (uNK) cells deplete their senescent counterparts.^46^, ^47^ After several days of inflammatory reprogramming, anti‐inflammatory decidual cells emerge that are highly resistant to metabolic and oxidative stress.^4^, ^48^ In clinical studies using ultrasonography, endometrial volume showed a constant increase as a result of an increase in estrogen level in the proliferative phase; however, the increase in endometrial thickness stopped during the secretory phase after ovulation.^49^ Recent reports have shown that a decrease in endometrial thickness after ovulation (endometrial compaction) is an important finding for successful pregnancy.^50^, ^51^ Considered together in the process of decidual change in HESCs, senescent decidual cells are eliminated and specialized anti‐inflammatory decidual cells emerge, leading to a slightly decreasing endometrial volume. During the secretory phase, PRP has a potential for promoting endometrial compaction with an anti‐inflammatory response, which supports successful pregnancy.

Decidual transformation of the endometrium and subsequent trophoblast invasion require downregulation of the PI3K/AKT signaling pathway.^31^, ^52^ PI3K is composed of two subunits: the catalytic subunit, p110, referred to as PIK3C, and an adaptor/regulatory subunit, p85, also referred to as PIK3R.^53^ PI3K enzymes primarily act to convert abundant cellular phosphatidylinositol‐4,5‐bisphosphate (PIP_2_) to phosphatidylinositol‐3,4,5‐triphosphate (PIP_3_), which mediates AKT phosphorylation and downstream signaling. Phosphatase and tensin homolog (PTEN) antagonizes PI3K‐dependent signaling via the dephosphorylation of PIP_3_ to PIP_2_.^54^ PTEN is abundantly expressed in decidualized stromal cells compared with undifferentiated cells; therefore, the increased PTEN diminishes the effects of PI3K signaling and regulates vascular remodeling through the infiltration of uNK cells during decidualization.^55^ In our results, PRP was shown to upregulate the expression level of *PTEN* and decrease the expression of the main subunits of PI3K, *PIK3CA* (*p100α*), and *PIK3R1* (*p85α*). Therefore, PRP mainly decreased the expression of PI3K by upregulating the expression of *PTEN*. In burn injury treatments, PRP also induced the expression of PTEN and suppressed the expression of PI3K/AKT and its related pathways, resulting in the alleviation of neuropathic pain.^11^ Furthermore, some reports showed that *TC2N* and *SERPINE2* (also known as *PN1*) in upregulated genes by PRP have suppressive effects on the PI3K/AKT signaling pathway,^56^, ^57^ and *RASGRP2* and *SPHK1* in downregulated genes were shown to regulate the PI3K/AKT signaling pathway.^58^, ^59^

Moreover, PRP downregulated the expression of toll‐like receptor 4 (*TLR4*) and its downstream genes *MAPK14* (also known as *p38*), DEP domain‐containing MTOR‐interacting protein (*DEPTOR*), and *Myc*. TLR4 is the integral receptor in the response to protect against the non‐self‐antigens, including microorganism infection with inflammatory response as a Th1‐dominant cell balance.^60^ PI3K signaling is also regulated via TLR4.^60^ Successful pregnancy requires maternal immune tolerance to a semiallogeneic embryo with a balance of proinflammatory cytokines and anti‐inflammatory cytokines, secreted by Th1 and Th2 cells, respectively, in favor of a Th2 dominance.^61^ RIF and recurrent pregnancy loss have been linked to an aberrantly high Th1/Th2 cell ratio.^62^ An infusion of PRP may regulate high Th1 response via a decrease in the expression of TLR4 signaling and improve endometrial immune tolerance for implantation in decidualized endometrium.

Platelet‐rich plasma treatment has many beneficial effects in terms of improving endometrial receptivity; however, PRP diminished the expression of decidual marker genes. One of the markers, IGFBP1, is predominantly produced by senescent decidual cells in primary cultures.^47^ It may result from a suppressive effect of PRP on cell proliferation of senescent cells, yet PRP may inhibit the decidualization of HESCs, resulting in a detrimental effect on pregnancy outcomes in unselected women who do not have an impaired endometrial environment. In previous clinical trials that did not consider the inclusion criteria of endometrial status, the therapeutic effect of intrauterine PRP infusion was not recognized in IVF treatment.^63^, ^64^

There were some limitations to this study. First, endometrial and blood samples were collected from the same women; therefore, given the limited number of extracted RNA samples, only microarray analysis and PCR array of undifferentiated and decidualized HESCs treated with and without PRP were performed, to identify detailed PRP‐dependent genes of the PI3K‐AKT signaling pathway. Second, the 10% of PRP concentration in the culture media was determined based on 1 ml of PRP and approximately 10 cm^3^ of an average intrauterine volume in adult women. However, we could not conduct a dose‐finding study because of the limited samples. Third, the blood sample was kept in the transfusion pack in cold storage for 4–6 days. The anticoagulant, CPDA, contributes to maintaining the function and viability of the platelets; however, according to a previous report,^65^ platelet count decreased by 20%–30% after 4–6 days of storage in the CPDA‐containing transfusion pack. Therefore, storage in a blood transfusion pack may affect the blood components and PRP. Fourth, the effect of PRP on endometrial epithelial cells was not analyzed in this study.

**FIGURE 4:**
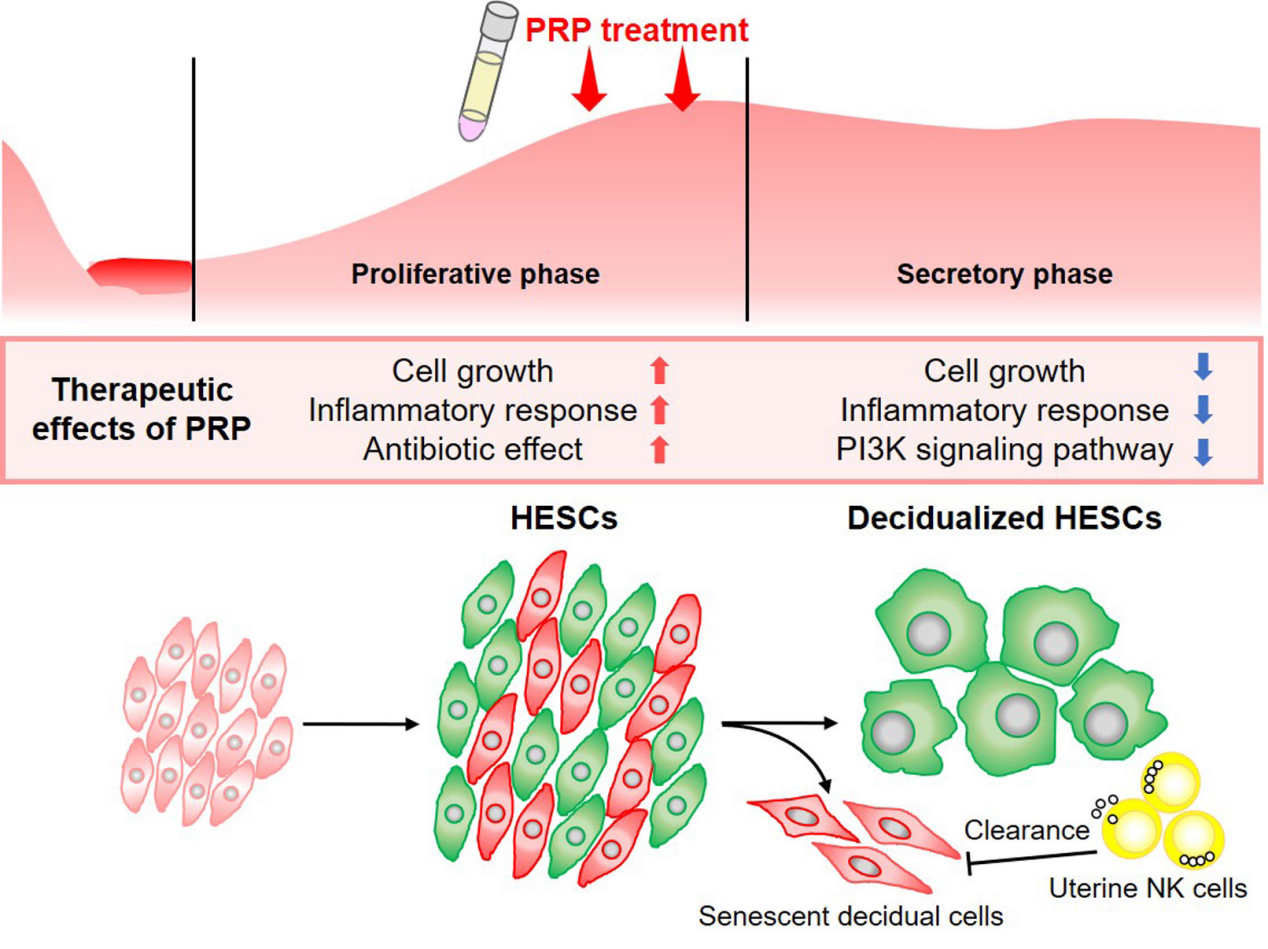
Therapeutic effects of intrauterine platelet‐rich plasma (PRP) infusion in proliferative and secretory phases. Intrauterine PRP infusion promotes tissue repair with transient inflammatory response, cell growth and migration, and antibiotic effect in undifferentiated human endometrial stromal cells, whereas cell proliferation and the inflammatory immune response are attenuated during decidualization of the endometrium. Depending on the menstrual cycle phases, PRP can regulate the reprogramming of inflammation and cell proliferation in an appropriate manner to support embryo implantation.

In summary, our observations suggest that intrauterine PRP infusion promotes tissue repair with transient inflammatory response, cell growth, and antimicrobial effect in undifferentiated HESCs, whereas cell proliferation and the inflammatory immune response are attenuated during decidualization of the endometrium (Figure 4). Depending on the menstrual cycle phases, PRP can regulate the reprogramming of inflammation and cell proliferation in an appropriate manner to support embryo implantation. However, PRP may have a suppressive effect on the decidual transformation of endometrium; thus, it may benefit only infertile women who have impaired intrauterine environment. Further investigation is required to elucidate the mechanism of action of PRP on the human endometrium.

## CONFLICT OF INTEREST

All authors have no conflicts of interest to declare relevant to this study.

## ETHICAL APPROVAL

This study was approved by the local ethics committee of Juntendo University, Faculty of Medicine (No. 20‐178) and Sugiyama Clinic (No. 19‐005). All procedures followed were in accordance with the ethical standards of the responsible committee on human experimentation and with the Helsinki Declaration of 1964 and its later amendments.

## INFORMED CONSENT

All recruited women provided written informed consent.

## ANIMAL STUDIES

This article does not contain any study with animal participants that have been performed by any of the authors.

## Supporting information


Figure S1.

Table S1.
Table S2.
Table S3.
Table S4.

## Data Availability

The data that support the findings of this study are available on request from the corresponding author. The microarray data sets used in the present study were deposited in the Gene Expression Omnibus under the accession number of GSE213873 (https://www.ncbi.nlm.nih.gov/geo/query/acc.cgi?acc=GSE213873).

## References

[1] Scott RT . Introduction: subchromosomal abnormalities in preimplantation embryonic aneuploidy screening. Fertil Steril. 2017;107(1):4–5. 28040094 10.1016/j.fertnstert.2016.11.017

[2] Treff NR , Franasiak JM . Detection of segmental aneuploidy and mosaicism in the human preimplantation embryo: technical considerations and limitations. Fertil Steril. 2017;107(1):27–31. 27816233 10.1016/j.fertnstert.2016.09.039

[3] Liu S , Diao L , Huang C , Li Y , Zeng Y , Kwak‐Kim JYH . The role of decidual immune cells on human pregnancy. J Reprod Immunol. 2017;124:44–53. 29055791 10.1016/j.jri.2017.10.045

[4] Gellersen B , Brosens JJ . Cyclic Decidualization of the human endometrium in reproductive health and failure. Endocr Rev. 2014;35(6):851–905. 25141152 10.1210/er.2014-1045

[5] Kasius A , Smit JG , Torrance HL , Eijkemans MJC , Mol BW , Opmeer BC , et al. Endometrial thickness and pregnancy rates after IVF: a systematic review and meta‐analysis. Hum Reprod Update. 2014;20(4):530–41. 24664156 10.1093/humupd/dmu011

[6] Chan RW , Schwab KE , Gargett CE . Clonogenicity of human endometrial epithelial and stromal cells. Biol Reprod. 2004;70(6):1738–50. 14766732 10.1095/biolreprod.103.024109

[7] Gargett CE , Ye L . Endometrial reconstruction from stem cells. Fertil Steril. 2012;98(1):11–20. 22657248 10.1016/j.fertnstert.2012.05.004

[8] Gleicher N , Kim A , Michaeli T , Lee HJ , Shohat‐Tal A , Lazzaroni E , et al. A pilot cohort study of granulocyte colony‐stimulating factor in the treatment of unresponsive thin endometrium resistant to standard therapies. Hum Reprod. 2013;28(1):172–7. 23081869 10.1093/humrep/des370

[9] Hou X , Liu Y , Streuli I , Dällenbach P , Dubuisson J , Ansaldi Y , et al. Endometrial regeneration in Asherman's syndrome: clinical and translational evidence of stem cell therapies. Curr Stem Cell Res Ther. 2019;14(6):454–9. 30760192 10.2174/1574888X14666190213100528

[10] Li J , Mo S , Chen Y . The effect of G‐CSF on infertile women undergoing IVF treatment: a meta‐analysis. Syst Biol Reprod Med. 2017;63(4):239–47. 28632452 10.1080/19396368.2017.1287225

[11] Huang SH , Wu SH , Lee SS , Lin YN , Chai CY , Lai CS , et al. Platelet‐rich plasma injection in burn scar areas alleviates neuropathic scar pain. Int J Med Sci. 2018;15(3):238–47. 29483815 10.7150/ijms.22563 PMC5820853

[12] Saita Y , Kobayashi Y , Nishio H , Wakayama T , Fukusato S , Uchino S , et al. Predictors of effectiveness of platelet‐rich plasma therapy for knee osteoarthritis: a retrospective cohort study. J Clin Med. 2021;10(19):4514. 34640529 10.3390/jcm10194514 PMC8509123

[13] Anitua E , Muruzabal F , de la Fuente M , Merayo J , Durán J , Orive G . Plasma rich in growth factors for the treatment of ocular surface diseases. Curr Eye Res. 2016;41(7):875–82. 26828610 10.3109/02713683.2015.1104362

[14] Gurtner GC , Werner S , Barrandon Y , Longaker MT . Wound repair and regeneration. Nature. 2008;453(7193):314–21. 18480812 10.1038/nature07039

[15] Gawaz M , Langer H , May AE . Platelets in inflammation and atherogenesis. J Clin Invest. 2005;115(12):3378–84. 16322783 10.1172/JCI27196 PMC1297269

[16] Stellos K , Kopf S , Paul A , Marquardt J , Gawaz M , Huard J , et al. Platelets in regeneration. Semin Thromb Hemost. 2010;36(2):175–84. 20414833 10.1055/s-0030-1251502

[17] Fabbro MD , Bortolin M , Taschieri S , Ceci C , Weinstein RL . Antimicrobial properties of platelet‐rich preparations. A systematic review of the current pre‐clinical evidence. Platelets. 2016;27(4):276–85. 26763769 10.3109/09537104.2015.1116686

[18] Hajipour H , Farzadi L , Latifi Z , Keyhanvar N , Navali N , Fattahi A , et al. An update on platelet‐rich plasma (PRP) therapy in endometrium and ovary related infertilities: clinical and molecular aspects. Syst Biol Reprod Med. 2021;67(3):177–88. 33632047 10.1080/19396368.2020.1862357

[19] Eftekhar M , Neghab N , Naghshineh E , Khani P . Can autologous platelet rich plasma expand endometrial thickness and improve pregnancy rate during frozen‐thawed embryo transfer cycle? A randomized clinical trial. Taiwan J Obstet Gynecol. 2018;57(6):810–3. 30545532 10.1016/j.tjog.2018.10.007

[20] Kusumi M , Ihana T , Kurosawa T , Ohashi Y , Tsutsumi O . Intrauterine administration of platelet‐rich plasma improves embryo implantation by increasing the endometrial thickness in women with repeated implantation failure: a single‐arm self‐controlled trial. Reprod Med Biol. 2020;19(4):350–6. 33071636 10.1002/rmb2.12334 PMC7542012

[21] Zamaniyan M , Peyvandi S , Heidaryan Gorji H , Moradi S , Jamal J , Yahya Poor Aghmashhadi F , et al. Effect of platelet‐rich plasma on pregnancy outcomes in infertile women with recurrent implantation failure: a randomized controlled trial. Gynecol Endocrinol. 2021;37(2):141–5. 32363968 10.1080/09513590.2020.1756247

[22] Coksuer H , Akdemir Y , Ulas BM . Improved in vitro fertilization success and pregnancy outcome with autologous platelet‐rich plasma treatment in unexplained infertility patients that had repeated implantation failure history. Gynecol Endocrinol. 2019;35(9):815–8. 30966843 10.1080/09513590.2019.1597344

[23] Chang Y , Li J , Chen Y , et al. Autologous platelet‐rich plasma promotes endometrial growth and improves pregnancy outcome during in vitro fertilization. Int J Clin Exp Med. 2015;8(1):1286–90. 25785127 PMC4358582

[24] Zadehmodarres S , Salehpour S , Saharkhiz N , Nazari L . Treatment of thin endometrium with autologous platelet‐rich plasma: a pilot study. JBRA Assist Reprod. 2017;21(1):54–6. 28333034 10.5935/1518-0557.20170013 PMC5365202

[25] Molina A , Sánchez J , Sánchez W , Vielma V . Platelet‐rich plasma as an adjuvant in the endometrial preparation of patients with refractory endometrium. JBRA Assist Reprod. 2018;22(1):42–8. 29303234 10.5935/1518-0557.20180009 PMC5844658

[26] Kuroda K , Matsumura Y , Ikemoto Y , Segawa T , Hashimoto T , Fukuda J , et al. Analysis of the risk factors and treatment for repeated implantation failure: OPtimization of thyroid function, IMmunity and uterine milieu (OPTIMUM) treatment strategy. Am J Reprod Immunol. 2020;85:e13376. 33166020 10.1111/aji.13376

[27] Kuroda K , Ikemoto Y , Horikawa T , Moriyama A , Ojiro Y , Takamizawa S , et al. Novel approaches to the management of recurrent pregnancy loss: the OPTIMUM (OPtimization of thyroid function, thrombophilia, immunity, and uterine milieu) treatment strategy. Reprod Med Biol. 2021;20(4):524–36. 34646081 10.1002/rmb2.12412 PMC8499598

[28] Ozaki R , Kuroda K , Ikemoto Y , Ochiai A , Matsumoto A , Kumakiri J , et al. Reprogramming of the retinoic acid pathway in decidualizing human endometrial stromal cells. PLoS One. 2017;12(3):e0173035. 28253328 10.1371/journal.pone.0173035 PMC5333850

[29] Barros FSV , Brosens JJ , Brighton PJ . Isolation and primary culture of various cell types from whole human endometrial biopsies. Bio‐protocol. 2016;6(22):e2028.

[30] Hagen CP , Mouritsen A , Mieritz MG , Tinggaard J , Wohlfahrt‐Veje C , Fallentin E , et al. Uterine volume and endometrial thickness in healthy girls evaluated by ultrasound (3‐dimensional) and magnetic resonance imaging. Fertil Steril. 2015;104(2):452–459.e452. 26051091 10.1016/j.fertnstert.2015.04.042

[31] Fabi F , Grenier K , Parent S , Adam P , Tardif L , Leblanc V , et al. Regulation of the PI3K/Akt pathway during decidualization of endometrial stromal cells. PLoS One. 2017;12(5):e0177387. 28475617 10.1371/journal.pone.0177387 PMC5419658

[32] Aghajanova L , Houshdaran S , Balayan S , Manvelyan E , Irwin JC , Huddleston HG , et al. In vitro evidence that platelet‐rich plasma stimulates cellular processes involved in endometrial regeneration. J Assist Reprod Genet. 2018;35(5):757–70. 29404863 10.1007/s10815-018-1130-8 PMC5984879

[33] Amable PR , Carias RB , Teixeira MV , et al. Platelet‐rich plasma preparation for regenerative medicine: optimization and quantification of cytokines and growth factors. Stem Cell Res Ther. 2013;4(3):67. 23759113 10.1186/scrt218 PMC3706762

[34] Gujral P , Mahajan V , Lissaman AC , Ponnampalam AP . Histone acetylation and the role of histone deacetylases in normal cyclic endometrium. Reprod Biol Endocrinol. 2020;18(1):84. 32791974 10.1186/s12958-020-00637-5 PMC7425564

[35] Munro SK , Farquhar CM , Mitchell MD , Ponnampalam AP . Epigenetic regulation of endometrium during the menstrual cycle. Mol Hum Reprod. 2010;16(5):297–310. 20139117 10.1093/molehr/gaq010

[36] Kim JH , Park M , Paek JY , Lee WS , Song H , Lyu SW . Intrauterine infusion of human platelet‐rich plasma improves endometrial regeneration and pregnancy outcomes in a murine model of Asherman's syndrome. Front Physiol. 2020;11:105. 32116803 10.3389/fphys.2020.00105 PMC7033504

[37] Aghajanova L , Sundaram V , Kao CN , Letourneau JM , Manvelyan E , Cedars MI , et al. Autologous platelet‐rich plasma treatment for moderate‐severe Asherman syndrome: the first experience. J Assist Reprod Genet. 2021;38(11):2955–63. 34613578 10.1007/s10815-021-02328-5 PMC8609080

[38] Javaheri A , Kianfar K , Pourmasumi S , Eftekhar M . Platelet‐rich plasma in the management of Asherman's syndrome: an RCT. Int J Reprod Biomed. 2020;18(2):113–20. 32259005 10.18502/ijrm.v18i2.6423 PMC7097166

[39] Aghajanova L , Cedars MI , Huddleston HG . Platelet‐rich plasma in the management of Asherman syndrome: case report. J Assist Reprod Genet. 2018;35(5):771–5. 29455274 10.1007/s10815-018-1135-3 PMC5984883

[40] Garcia KC , Degano M , Speir JA , Wilson IA . Emerging principles for T cell receptor recognition of antigen in cellular immunity. Rev Immunogenet. 1999;1(1):75–90. 11256574

[41] Kimura F , Takebayashi A , Ishida M , Nakamura A , Kitazawa J , Morimune A , et al. Review: chronic endometritis and its effect on reproduction. J Obstet Gynaecol Res. 2019;45(5):951–60. 30843321 10.1111/jog.13937

[42] Kitaya K , Matsubayashi H , Takaya Y , Nishiyama R , Yamaguchi K , Takeuchi T , et al. Live birth rate following oral antibiotic treatment for chronic endometritis in infertile women with repeated implantation failure. Am J Reprod Immunol. 2017;78(5):e12719. 10.1111/aji.12719 28608596

[43] Davies SC , Fowler T , Watson J , Livermore DM , Walker D . Annual report of the chief medical officer: infection and the rise of antimicrobial resistance. Lancet. 2013;381(9878):1606–9. 23489756 10.1016/S0140-6736(13)60604-2

[44] Li F , Cui Y , Zhao D , Bao H , Hao C . Outcome study of five cases receiving in vitro fertilization after treatment of intrauterine platelet‐rich plasma (PRP) for chronic endometritis. Panminerva Med. 2021. doi:10.23736/S0031-0808.20.04247-0 33470585

[45] Sfakianoudis K , Simopoulou M , Nitsos N , et al. Successful implantation and live birth following autologous platelet‐rich plasma treatment for a patient with recurrent implantation failure and chronic endometritis. In Vivo. 2019;33(2):515–21. 30804135 10.21873/invivo.11504 PMC6506282

[46] Brighton PJ , Maruyama Y , Fishwick K , Vrljicak P , Tewary S , Fujihara R , et al. Clearance of senescent decidual cells by uterine natural killer cells in cycling human endometrium. Elife. 2017;6:e31274. 29227245 10.7554/eLife.31274 PMC5724991

[47] Lucas ES , Vrljicak P , Muter J , Diniz‐da‐Costa MM , Brighton PJ , Kong CS , et al. Recurrent pregnancy loss is associated with a pro‐senescent decidual response during the peri‐implantation window. Commun Biol. 2020;3(1):37. 31965050 10.1038/s42003-020-0763-1 PMC6972755

[48] Brosens JJ , Salker MS , Teklenburg G , Nautiyal J , Salter S , Lucas ES , et al. Uterine selection of human embryos at implantation. Sci Rep. 2014;4:3894. 24503642 10.1038/srep03894 PMC3915549

[49] Jokubkiene L , Sladkevicius P , Rovas L , Valentin L . Assessment of changes in endometrial and subendometrial volume and vascularity during the normal menstrual cycle using three‐dimensional power Doppler ultrasound. Ultrasound Obstet Gynecol. 2006;27(6):672–9. 16676367 10.1002/uog.2742

[50] Haas J , Smith R , Zilberberg E , Nayot D , Meriano J , Barzilay E , et al. Endometrial compaction (decreased thickness) in response to progesterone results in optimal pregnancy outcome in frozen‐thawed embryo transfers. Fertil Steril. 2019;112(3):503–509.e501. 31248618 10.1016/j.fertnstert.2019.05.001

[51] Zilberberg E , Smith R , Nayot D , Haas J , Meriano J , Barzilay E , et al. Endometrial compaction before frozen euploid embryo transfer improves ongoing pregnancy rates. Fertil Steril. 2020;113(5):990–5. 32386621 10.1016/j.fertnstert.2019.12.030

[52] Laguë MN , Detmar J , Paquet M , Boyer A , Richards JAS , Adamson SL , et al. Decidual PTEN expression is required for trophoblast invasion in the mouse. Am J Physiol Endocrinol Metab. 2010;299(6):E936–46. 20858757 10.1152/ajpendo.00255.2010 PMC3006249

[53] Fruman DA , Chiu H , Hopkins BD , Bagrodia S , Cantley LC , Abraham RT . The PI3K pathway in human disease. Cell. 2017;170(4):605–35. 28802037 10.1016/j.cell.2017.07.029 PMC5726441

[54] Cantley LC , Neel BG . New insights into tumor suppression: PTEN suppresses tumor formation by restraining the phosphoinositide 3‐kinase/AKT pathway. Proc Natl Acad Sci USA. 1999;96(8):4240–5. 10200246 10.1073/pnas.96.8.4240 PMC33561

[55] Mutter GL , Lin MC , Fitzgerald JT , Kum JB , Eng C . Changes in endometrial PTEN expression throughout the human menstrual cycle. J Clin Endocrinol Metab. 2000;85(6):2334–8. 10852473 10.1210/jcem.85.6.6652

[56] Hao XL , Gao LY , Deng XJ , Han F , Chen HQ , Jiang X , et al. Identification of TC2N as a novel promising suppressor of PI3K‐AKT signaling in breast cancer. Cell Death Dis. 2019;10(6):424. 31142739 10.1038/s41419-019-1663-5 PMC6541591

[57] McKee CM , Ding Y , Zhou J , et al. Protease nexin 1 induces apoptosis of prostate tumor cells through inhibition of X‐chromosome‐linked inhibitor of apoptosis protein. Oncotarget. 2015;6(6):3784–96. 25686839 10.18632/oncotarget.2921 PMC4414153

[58] Takino JI , Sato T , Nagamine K , Hori T . The inhibition of Bax activation‐induced apoptosis by RasGRP2 via R‐Ras‐PI3K‐Akt signaling pathway in the endothelial cells. Sci Rep. 2019;9(1):16717. 31723205 10.1038/s41598-019-53419-4 PMC6854084

[59] Quint P , Ruan M , Pederson L , Kassem M , Westendorf JJ , Khosla S , et al. Sphingosine 1‐phosphate (S1P) receptors 1 and 2 coordinately induce mesenchymal cell migration through S1P activation of complementary kinase pathways. J Biol Chem. 2013;288(8):5398–406. 23300082 10.1074/jbc.M112.413583 PMC3581421

[60] Fukao T , Koyasu S . PI3K and negative regulation of TLR signaling. Trends Immunol. 2003;24(7):358–63. 12860525 10.1016/s1471-4906(03)00139-x

[61] Ng SC , Gilman‐Sachs A , Thaker P , Beaman KD , Beer AE , Kwak‐Kim J . Expression of intracellular Th1 and Th2 cytokines in women with recurrent spontaneous abortion, implantation failures after IVF/ET or normal pregnancy. Am J Reprod Immunol. 2002;48(2):77–86. 12389596 10.1034/j.1600-0897.2002.01105.x

[62] Kuroda K , Nakagawa K , Horikawa T , et al. Increasing number of implantation failures and pregnancy losses associated with elevated Th1/Th2 cell ratio. Am J Reprod Immunol. 2021;86:e13429. 33835626 10.1111/aji.13429

[63] Madhavan A , Naidu P , Rani K , Kaur J , Mahajan N . Intrauterine autologous platelet‐rich plasma therapy to improve implantation rates in patients undergoing frozen embryo transfer: a pilot study. Onco Fertil J. 2018;1(2):81–5.

[64] Allahveisi A , Seyedoshohadaei F , Rezaei M , Bazrafshan N , Rahimi K . The effect of platelet‐rich plasma on the achievement of pregnancy during frozen embryo transfer in women with a history of failed implantation. Heliyon. 2020;6(3):e03577. 32195397 10.1016/j.heliyon.2020.e03577 PMC7075971

[65] Meledeo MA , Peltier GC , McIntosh CS , Bynum JA , Cap AP . Optimizing whole blood storage: hemostatic function of 35‐day stored product in CPD, CP2D, and CPDA‐1 anticoagulants. Transfusion. 2019;59(S2):1549–59. 30980756 10.1111/trf.15164

