# Supplementary material for: Transcriptomic profiling analysis of human endometrial stromal cells treated with autologous platelet‐rich plasma
Source: Reprod Med Biol. 2023 Jan 22;22(1):e12498. doi: 10.1002/rmb2.12498 (PMC9868347; doi:10.1002/rmb2.12498)
Supplement: Supplementary file 1 — Figure S1. [file RMB2-22-e12498-s001.docx]

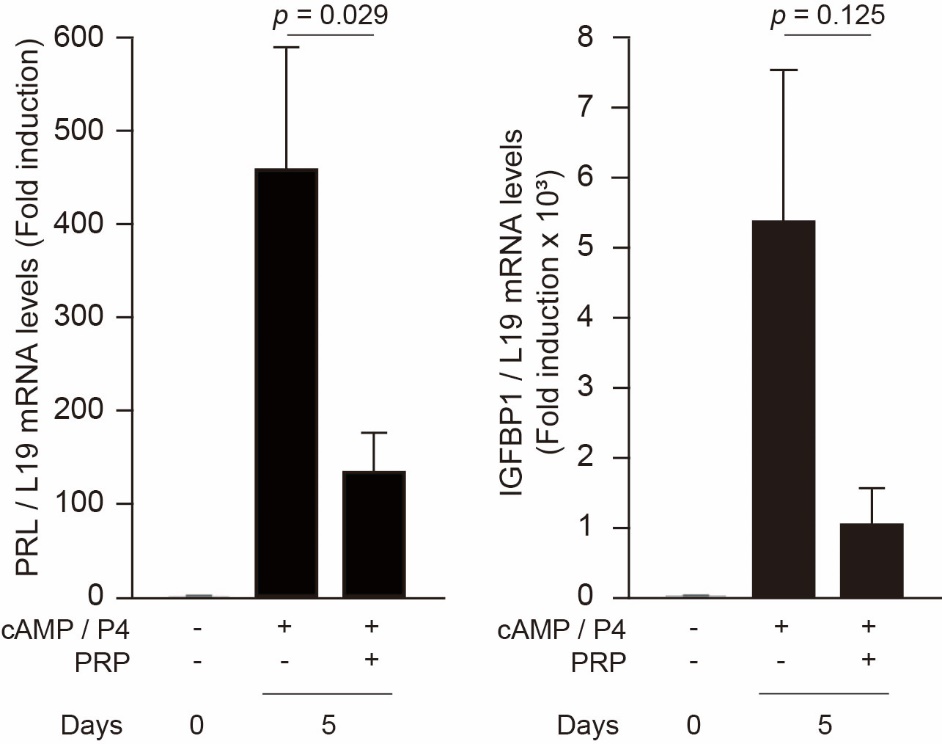


**Supplementary Figure 1. Platelet-rich plasma (PRP) attenuated the expression of decidual marker genes in decidualized human endometrial stromal cells** **(HESCs).** The induction of PRL and IGFBP1 mRNA in four independent primary cultures first treated with and without PRP twice and then treated with 8-bromo-cAMP and P4 for 5 days. Transcript levels were measured by real-time quantitative polymerase chain reaction (RTQ-PCR) and expressed as mean fold change ± SEM.
