# Supplementary material for: Transcriptomic profiling analysis of human endometrial stromal cells treated with autologous platelet‐rich plasma
Source: Reprod Med Biol. 2023 Jan 22;22(1):e12498. doi: 10.1002/rmb2.12498 (PMC9868347; doi:10.1002/rmb2.12498)
Supplement: Supplementary file 2 — Table S1. Table S2. Table S3. Table S4. [file RMB2-22-e12498-s002.docx]

| **Table S1. Patient characteristics** | |
| --- | --- |
|  | **Infertile women, n = 4** |
| **Age, years, mean ± SD (range)** | 35.3 ± 5.2 (29–40) |
| **Pregnancy history, median (range)**  Gravida  Parity | 1 (0–4)  0 (0–1) |
| **Duration of infertility, years, mean ± SD** | 2.9 ± 1.1 |
| **Serum AMH level, ng/ml, mean ± SD** | 3.6 ± 2.1 |
| **Causes for infertility, n (%)**  **Tubal factor**  **Unexplained infertility** | 2 (50.0)  2 (50.0) |
| **No. of implantation failure after ET, median (range)**  Cleavage stage embryos, median (range)  Blastocysts, median (range)  Number of transferred embryos per cycle, mean ± SD | 4 (4–5)  0.5 (0–1)  3.5 (3–5)  1.0 ± 0 |
| **No. of CD138-positive plasma cells^†^, mean ± SD** | 1.0 ± 1.4 |
| **Laboratory data, mean ± SD**  White blood cell count, /μL  Red blood cell count, 10^4^/μL  Hemoglobin, g/dL  Hematocrit, %  Platelet count, 10^4^/μL | 5435.0 ± 1109.7  406.5 ± 14.4  12.7 ± 0.3  38.2 ± 1.0  24.1 ± 3.6 |

SD = standard deviation

AMH = anti-Müllerian hormone

ET = embryo transfer

^†^ Immunohistochemistry for plasma cells (CD138) was performed within 3 months before or after the ERA test and counted in 10 nonoverlapping random stromal areas visualized at 400-fold magnification.

| **Table S2. Genes regulated by PRP treatment in both undifferentiated and decidualized HESCs** | | | |
| --- | --- | --- | --- |
| **PRP affected genes in undifferentiated HESCs** | **PRP affected genes in decidualized HESCs** | **Decidualization affected genes** | **Number of genes** |
| **Up** | **–** | **–** | 86 |
| **Down** | **–** | **–** | 154 |
| **–** | **Up** | **–** | 13 |
| **–** | **Down** | **–** | 8 |
| **–** | **–** | **Up** | 353 |
| **–** | **–** | **Down** | 412 |
| **Up** | **Up** | **–** | 2 |
| **Down** | **Up** | **–** | 1 |
| **Down** | **Down** | **–** | 1 |
| **Up** | **–** | **Up** | 9 |
| **Up** | **–** | **Down** | 49 |
| **Down** | **–** | **Up** | 33 |
| **Down** | **–** | **Down** | 40 |
| **–** | **Up** | **Up** | 1 |
| **–** | **Up** | **Down** | 1 |
| **–** | **Down** | **Up** | 30 |
| **Up** | **Down** | **Up** | 1 |
| **Down** | **Down** | **Up** | 4 |
| **Down** | **Down** | **Down** | 1 |

| **Table S3. Genes regulated by PRP treatment in both undifferentiated and decidualized HESCs** | | | | |
| --- | --- | --- | --- | --- |
| **Gene symbol** | **Gene name** | **Undifferentiated HESCs treated with PRP**  **fold change** | **Decidualized HESCs treated with PRP**  **fold change** | **Decidualization**  **fold change** |
| TRHDE  CST4  SRGN  SULT1C4  OLFML2B  ACKR3  TLR4  ECM2  MGP  CFH | thyrotropin-releasing hormone degrading enzyme  cystatin S  serglycin  sulfotransferase family 1C member 4  olfactomedin like 2B  atypical chemokine receptor 3  toll-like receptor 4  extracellular matrix protein 2  matrix Gla protein  complement factor H | 4.31  3.04  2.50  -1.38  -1.06  -1.29  -1.48  -1.55  -1.61  -2.16 | 2.10  2.31  -1.16  2.73  -1.07  -1.13  -1.79  -1.35  -1.54  -1.96 | –  –  5.61  –  2.36  –  2.22  3.57  -3.21  7.76 |

| **Table S4. PRP affected genes in the PI3K/AKT signaling pathway in decidualized HESCs** | | | |
| --- | --- | --- | --- |
| **Gene symbol** | **Gene name** | **fold change^†^** | **p-value** |
| TLR4  CASP9  LYN  MYC  FYN  PIK3R1 (p85-alpha)  MAPK14  SGK1  SPP1  PIK3CA (p110-alpha)  PDPK1  GRB2  MAP3K5  RAF1  MAPK3  SRC  IGF1R  JAK2  KIT  ABL1  SH3KBP1  ERBB2  BCL2L1  PRKCZ  RHOA  PTK2  GUSB  PIK3R4  CTNNB1  RAC1  GYS1  SHC1  PRKCA  PIK3CB  RELA  ARHGEF7  ITGB1  CDH1  PIK3C2A  MAPK1  KRAS  VAV1  AKT1  MTOR  MAP2K1  MAPK8  LCK  INPP5D  EGFR  GSK3B  BAD  NFKB1  HRAS  CD4  BCL2  NFKBIA  CDKN1A  PIK3C2B  E2F1  RPS6KB1  CDKN1B  IRAK4  IRS1  SOS1  PRKCD  FAS  CCND1  PLCG1  TEC  PAK1  CDC42  SYK  CD40  PIK3R2  HPRT1  CREB1  GNB3  CDKN2A  PIK3CD  ITGB3  PIK3C3 (Vps34)  PTEN  PRKCB  PDE3B  TNF  CXCR4 | toll-like receptor 4  caspase 9  LYN proto-oncogene, Src family tyrosine kinase  MYC proto-oncogene, bHLH transcription factor  FYN proto-oncogene, Src family tyrosine kinase  phosphoinositide-3-kinase regulatory subunit 1  mitogen-activated protein kinase 14  serum/glucocorticoid regulated kinase 1  secreted phosphoprotein 1  phosphatidylinositol-4,5-bisphosphate 3-kinase catalytic subunit alpha  3-phosphoinositide dependent protein kinase 1  growth factor receptor bound protein 2  mitogen-activated protein kinase kinase kinase 5  Raf-1 proto-oncogene, serine/threonine kinase  mitogen-activated protein kinase 3  SRC proto-oncogene, non-receptor tyrosine kinase  insulin like growth factor 1 receptor  Janus kinase 2  KIT proto-oncogene, receptor tyrosine kinase  ABL proto-oncogene 1, non-receptor tyrosine kinase  SH3 domain containing kinase binding protein 1  erb-b2 receptor tyrosine kinase 2  BCL2 like 1  protein kinase C zeta  ras homolog family member A  protein tyrosine kinase 2  glucuronidase beta  phosphoinositide-3-kinase regulatory subunit 4  catenin beta 1  Rac family small GTPase 1  glycogen synthase 1  SHC adaptor protein 1  protein kinase C alpha  phosphatidylinositol-4,5-bisphosphate 3-kinase catalytic subunit beta  RELA proto-oncogene, NF-kB subunit  Rho guanine nucleotide exchange factor 7  integrin subunit beta 1  cadherin 1  phosphatidylinositol-4-phosphate 3-kinase catalytic subunit type 2 alpha  mitogen-activated protein kinase 1  KRAS proto-oncogene, GTPase  vav guanine nucleotide exchange factor 1  AKT serine/threonine kinase 1  mechanistic target of rapamycin kinase  mitogen-activated protein kinase kinase 1  mitogen-activated protein kinase 8  LCK proto-oncogene, Src family tyrosine kinase  inositol polyphosphate-5-phosphatase D  epidermal growth factor receptor  glycogen synthase kinase 3 beta  BCL2 associated agonist of cell death  nuclear factor kappa B subunit 1  HRas proto-oncogene, GTPase  CD4 molecule  BCL2 apoptosis regulator  NFKB inhibitor alpha  cyclin dependent kinase inhibitor 1A  phosphatidylinositol-4-phosphate 3-kinase catalytic subunit type 2 beta  E2F transcription factor 1  ribosomal protein S6 kinase B1  cyclin dependent kinase inhibitor 1B  interleukin 1 receptor associated kinase 4  insulin receptor substrate 1  SOS Ras/Rac guanine nucleotide exchange factor 1  protein kinase C delta  Fas cell surface death receptor  cyclin D1  phospholipase C gamma 1  tec protein tyrosine kinase  p21 (RAC1) activated kinase 1  cell division cycle 42  spleen associated tyrosine kinase  CD40 molecule  phosphoinositide-3-kinase regulatory subunit 2  hypoxanthine phosphoribosyltransferase 1  cAMP responsive element binding protein 1  G protein subunit beta 3  cyclin dependent kinase inhibitor 2A  phosphatidylinositol-4,5-bisphosphate 3-kinase catalytic subunit delta  integrin subunit beta 3  phosphatidylinositol 3-kinase catalytic subunit type 3  phosphatase and tensin homolog  protein kinase C beta  phosphodiesterase 3B  tumor necrosis factor  C-X-C motif chemokine receptor 4 | 0.21 ± 0.05  0.28 ± 0.05  0.37 ± 0.09  0.37 ± 0.12  0.41 ± 0.12  0.49 ± 0.17  0.55 ± 0.06  0.56 ± 0.16  0.64 ± 0.14  0.73 ± 0.10  0.73 ± 0.11  0.75 ± 0.15  0.75 ± 0.15  0.77 ± 0.07  0.86 ± 0.09  0.87 ± 0.13  0.88 ± 0.12  0.88 ± 0.37  0.89 ± 0.13  0.91 ± 0.21  0.93 ± 0.07  0.93 ± 0.07  0.93 ± 0.07  0.93 ± 0.07  0.93 ± 0.08  0.98 ± 0.19  0.99 ± 0.19  1.00 ± 0.00  1.00 ± 0.00  1.00 ± 0.00  1.00 ± 0.00  1.00 ± 0.00  1.00 ± 0.00  1.00 ± 0.01  1.00 ± 0.01  1.00 ± 0.01  1.00 ± 0.01  1.00 ± 0.01  1.01 ± 0.01  1.01 ± 0.01  1.02 ± 0.14  1.03 ± 0.02  1.03 ± 0.15  1.04 ± 0.15  1.10 ± 0.10  1.10 ± 0.11  1.10 ± 0.18  1.10 ± 0.31  1.11 ± 0.10  1.11 ± 0.11  1.11 ± 0.11  1.12 ± 0.32  1.14 ± 0.18  1.14 ± 0.34  1.15 ± 0.29  1.18 ± 0.29  1.20 ± 0.12  1.20 ± 0.35  1.21 ± 0.11  1.21 ± 0.13  1.21 ± 0.13  1.24 ± 0.25  1.24 ± 0.26  1.26 ± 0.25  1.26 ± 0.26  1.26 ± 0.26  1.27 ± 0.27  1.30 ± 0.10  1.31 ± 0.10  1.34 ± 0.23  1.36 ± 0.23  1.36 ± 0.23  1.36 ± 0.24  1.36 ± 0.24  1.36 ± 0.25  1.37 ± 0.25  1.46 ± 0.20  1.47 ± 0.21  1.48 ± 0.21  1.51 ± 0.29  1.57 ± 0.15  1.61 ± 0.25  1.63 ± 0.49  1.75 ± 0.26  2.05 ± 0.48  2.44 ± 0.63 | 0.03  0.03  0.03  0.03  0.03  0.03  0.03  NS  0.03  0.03  NS  NS  NS  0.03  NS  NS  NS  NS  NS  NS  NS  NS  NS  NS  NS  NS  NS  NS  NS  NS  NS  NS  NS  NS  NS  NS  NS  NS  NS  NS  NS  NS  NS  NS  NS  NS  NS  NS  NS  NS  NS  NS  NS  NS  NS  NS  NS  NS  0.03  NS  NS  NS  NS  0.03  NS  NS  NS  0.03  0.03  NS  0.03  0.03  NS  NS  NS  NS  NS  NS  NS  NS  0.03  0.03  NS  NS  0.03  0.03 |

NS = no significant difference

**^†^**Fold changes are shown as mean ± standard error of the mean.
